# Human cytomegalovirus tegument proteins (pp65, pp71, pp150, pp28)

**DOI:** 10.1186/1743-422X-9-22

**Published:** 2012-01-17

**Authors:** John Paul Tomtishen III

**Affiliations:** 1 Bucknell University, Cell Biology/Biochemistry Program, One Dent Drive, Lewisburg, PA 17837, USA

**Keywords:** Human cytomegalovirus, HCMV, Tegument, pp65, pp71, pp150, pp28, Transfection, Confocal microscopy

## Abstract

Human cytomegalovirus (HCMV), a member of the *Betaherpesvirinae *sub-family of *Herpesviridae *family, is a widespread pathogen that infects a majority of the world's population by early adulthood. In individuals whose immune systems are immature or weakened, HCMV is a significant pathogen causing morbidity and mortality. There is no effective vaccine and only limited antiviral treatments against HCMV infection to date. A possible target for novel antiviral treatments is the HCMV proteins that localize to the tegument of the virion, since they play important roles in all stages of the viral life cycle, including, viral entry, gene expression, immune evasion, assembly, and egress. The most likely tegument protein candidates are pp65 (immune evasion), pp71 (gene expression), and pp150 and pp28 (assembly and egress). Although the subcellular localization of these proteins has been identified during HCMV infections in vitro, their localization patterns have not been determined when each protein is expressed individually in living cells. Thus, the objective of this review is elucidate the HCMV tegument as well as present current research findings concerning the subcellular localization of the tegument proteins pp65, pp71, pp150, and pp28 as fusions to one of several fluorescent proteins.

## Human cytomegalovirus significance

Human cytomegalovirus (HCMV) is a member of the *Betaherpesvirinae *sub-family of *Herpesviridae*. It is a widespread pathogen that infects a majority of the world's population by early adulthood [1]. In fact, by the age of 40, between 50 and 85% of adults are infected by HCMV [2]. The virus establishes a life-long infection with some cells being latently infected, a state where the virus has the ability to lie dormant within a cell, while others are persistently infected, where the infection cannot be cleared from an organism and there is intermittent shedding of infectious virions [3]. Immunocompetent individuals, who can develop a strong immune response, typically display no symptoms of infection [4]. However, in individuals whose immune systems are immature or weakened, such as organ transplant and AIDS patients, HCMV is a significant pathogen causing morbidity and mortality [5]. Symptoms in these individuals typically consist of spiking fever, leucopenia (decrease in white blood cells), malaise, hepatitis, pneumonia, gastrointestinal disease and/or retinitis (inflammation of the retina) [4]. HCMV is also responsible for approximately 8% of infectious mononucleosis cases [6] and is the leading viral cause of birth defects often causing deafness and mental retardation in the fetus if a woman is infected during pregnancy [7].

HCMV has been implicated in playing a role in inflammatory and proliferative diseases, including certain cardiovascular diseases and cancer [8]. Epidemiological and pathological studies have espoused a strong link between HCMV and atherosclerosis [8]. Several mechanisms have been proposed in which HCMV could influence the development of athersclerotic vascular abnormalities [9]. A proposed role of HCMV in the pathogenesis of atherosclerosis involves the reactivation of a latent HCMV infection followed by virus-induced enhancement of vascular inflammation and damage through smooth cell proliferation, uptake of low-density lipoproteins by smooth cells, neointimal formation (thickened arterial layer via cell migration and proliferation), and narrowing of the vessel lumen [9].

There is no effective vaccine against HCMV, and drugs that inhibit viral replication exist but are ineffective due to high toxicity, low bioavailability, and the development of drug-resistant virus strains [8,10]. The primary antiviral agents used to treat HCMV infections in patients with an impaired immune system are ganciclovir, foscarnet, and cidofovir [9]. These drugs have improved the survival and quality of life of immunocompromised individuals suffering from HCMV, but they are far from ideal due to major hematologic, renal, and neutropenia toxicity [9]. Therefore, the lack of an effective treatment for HCMV infections, especially in immunocompromised individuals, has resulted in intense study for the identification of proteins and processes that could be targeted by novel antiviral treatments [8].

## Human cytomegalovirus structure and life cycle

**Figure 1:**
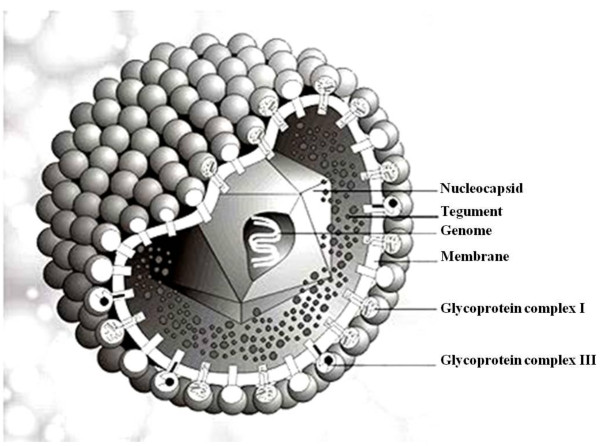
**A cartoon depicting the structure of the HCMV virion**. (Image obtained from (http://www.virology.net/big_virology/bvdnaherpes.html) courtesy of Dr. Marko Reschke in Marburg, Germany).

HCMV has the prototypical herpesvirus virion structure (Figure 1) and the replication cycle has a well controlled cascade of gene expression [9]. The virion has an icosahedral protein capsid that contains the 235-kb double-stranded DNA. The capsid is surrounded by a proteinaceous tegument and an outer lipid envelope [1]. Virions gain entry into a cell through a membrane fusion event involving the outer membrane of the cell and glycoproteins on the lipid envelope of virions. Once the fusion of these two membranes occurs, the DNA-containing protein capsid and the tegument proteins are released into the cell [11].

The gene expression pattern follows a similar cascade as used by other herpesviruses as reviewed in Kalejta (2008) [12]. During the lytic infection, viral immediate-early genes are expressed [8]. The expression of these genes results in the production of viral immediate-early proteins that modulate the host cell environment and stimulate the expression of viral early genes [1]. The viral-immediate early genes produce proteins that are responsible for replicating the double-stranded viral genomic DNA; after DNA replication, these immediate-early genes turn on the expression of viral late genes [1]. The viral late proteins are mainly structural components of the virion that assist in the assembly and egress of newly formed viral particles [1]. Immediate-early genes in HCMV can be silenced in certain cell types upon infection though, which results in a latent infection [13]. A latent infection is characterized by the minimization of viral gene expression and the inhibition of the assembly and egress of new viral progeny [11]. Latent infections can reactivate into a lytic infection upon certain environmental cues, which causes disease and allows viral spread [1,12].

HCMV infected cells also produce noninfectious enveloped particles and dense bodies in addition to infectious virions as reviewed in Kalejta (2008) [12]. Noninfectious enveloped particles are similar to infectious virions in that they contain a nearly an identical assortment of envelope, tegument, and capsid proteins, but they lack the double-stranded viral genome within the icosahedral capsid [14]. Dense bodies on the other hand are enveloped tegument proteins that lack capsids and are primarily composed of the viral pp65 protein [14]. The significance of the noninfectious enveloped particles and dense bodies is not known in wild-type strains of HCMV [14].

## Tegument structure and function

A possible target for novel antiviral treatments is the HCMV proteins that localize to the tegument. As mentioned, the tegument in HCMV is located between the outer lipid membrane and the icosahedral protein capsid, which contains the viral genomic double-stranded DNA [11]. The tegument is generally thought to be unstructured and amorphous in nature although some structuring is seen with the binding of tegument proteins to the protein capsid [15]. The tegument proteins comprise more than half of the total proteins found within infectious virions [16]. Tegument proteins are phosphorylated, but the significance of this and other posttranslational modifications to these proteins remains largely unexplored [11]. A common sequence to direct proteins into the tegument has not been identified through either experimental or bioinformatic approaches [17]. The process of assembling the viral tegument upon viral egress and disassembly upon viral entry into cells is largely unknown [17]. However as reviewed in Kalejta (2008), phosphorylation, subcellular localization to the assembly site, and interaction with capsids or the cytoplasmic tails of envelope proteins, likely facilitate the incorporation of proteins into the HCMV tegument [12].

As mentioned above, virions gain entry into a cell through a membrane fusion event involving the outer membrane of the cell and the lipid membrane of virions. The entry of the tegument proteins as well as the DNA-containing protein capsid upon viral entry occurs after the fusion of these two membranes [11]. Upon release into the cytoplasm, tegument proteins become functionally active, where they play important roles in all stages of the viral life cycle, including, viral entry, gene expression, immune evasion, assembly, and egress [1,11]. There are several tegument proteins that are of particular interest due to the role (elucidated below) that they play in the HCMV replication cycle, including pp65, pp71, pp150, and pp28.

## Tegument protein pp65

Pp65 is the most abundant tegument protein and the major constituent of extracellular virus particles [18]. However, pp65 is not essential for the production of new infectious virus particles as evidenced in strains that lack the pp65 gene [18] which can still replicate in culture. Pp65 is the major tegument protein responsible for modulating/evading the host cell immune response during HCMV infections [19]. As reviewed in Kalejta (2008), pp65 is implicated in counteracting both innate and adaptive immune responses during HCMV infections [12]. pp65's role in immune evasion is largely attributable to its targeting of both humoral and cellular immunity as well as serving as the dominant target antigen of cytotoxic T lymphocytes [19]. It has been demonstrated that pp65 not only prevents immediate-early proteins from being recognized by components of the immune system, but it also inhibits the synthesis of the various components involved in the host cell's immune response [20]. One of the ways in which pp65 counteracts adaptive immunity is through its enzymatic kinase activity [21]. It was shown that pp65 mediates the phosphorylation of viral immediate-early proteins, which blocks their presentation to the major histocompatibility complex class I molecules [22]. The kinase activity of pp65 has also been implicated in causing the degradation of the alpha chain in the major histocompatibility class II cell surface receptor, HLA-DR, via an accumulation of HLA class II molecules in the lysosome [20]. Furthermore, several studies have presented evidence that pp65 is involved in mediating a decrease in the expression of major histocompatibility complex class II molecules [20]. This is significant in that major histocompatibility complex class I and II molecules are responsible for lymphocyte recognition and antigen presentation with class I molecules presenting to cytotoxic T lymphocytes CD8+ and class II molecules to helper T lymphocytes CD4+ [20].

Another pivotal role that pp65 has in immune evasion during HCMV infections is through the inhibition of natural killer cell cytotoxicity [23]. Specifically, it was shown that pp65 can act as an antagonistic ligand that can bind to the NKp30 activating receptor to protect the killing of infected cells as well as interfere with the ability of NKp30 to cross-talk between other natural killer cells and dendritic cells [23,24].

Finally, pp65 has been shown to attenuate the interferon response [25]. It is thought that pp65 is involved in down modulating beta interferon and a number of chemokines, which is based on the observation of an elevated expression of interferon genes in infections with a strain lacking the pp65 protein [25].

Thus, the role that pp65 has in immune evasion in HCMV infections is to prevent infected cells from being destroyed by the immune system. Furthermore, it has been shown to protect infected cells from the immune response by binding to components of the immune system, thereby inhibiting their activation [24].

## Tegument protein pp71

Pp71, by comparison, plays an important role in the activation of immediate-early gene expression at the start of the lytic replication cycle [26]. Although this protein is not absolutely essential, it is necessary for efficient viral replication as reviewed in Kalejta (2008) [12]. A proposed mechanism for how pp71 activates viral gene expression is by neutralizing the effects of the cellular Daxx protein, which is recruited to promoters by DNA-binding transcription factors, resulting in the repression of transcription [27]. Pp71 can bind to two inherent domains on Daxx and induce its proteasomal degradation [28]. Additionally, it was demonstrated that pp71 increases the infectivity of viral genomic DNA when transfected into cultured cells [29].

Recently though, pp71 has also been implicated in immune evasion, similar to pp65, by disrupting the major cell surface expression of components of the immune response [30]. Specifically, pp71 appears to target the cell surface receptors of major histocompatibility complex class I proteins by slowing their intracellular transport [30]. This limits the ability of infected cells to display viral antigens to the immune system and prevents recognition by cytotoxic T lymphocytes.

## Tegument proteins pp150 and pp28

Pp150 and pp28 are highly immunogenic and play roles in the assembly and egress of virus particles. Both of these tegument proteins play very similar roles, but have some distinct functions. pp150, the second most abundant tegument protein behind pp65, is necessary to incorporate nucleocapsids into virus particles [16]. Pp150 is essential for maintaining the stability of the cytoplasmic capsids and directing their movement [1,31].

Pp150 also plays a role in the reorganization of the cytoplasmic assembly compartment during virion assembly [31]. The process of virion assembly in HCMV has been reviewed in Kalejta (2008) [12]. After the viral genome and late genes are expressed, capsid formation and DNA packaging into the preformed capsids begins to occur in the nucleus. Capsids acquire a primary envelope when they bud through the inner nuclear membrane into the perinuclear space, which they lose upon budding through the outer nuclear membrane into the cytoplasm. The capsids then bud into Golgi apparatus-derived vesicles and obtain their final envelope. When these vesicles fuse with the cell membrane, the enveloped virion is released. Pp28 is largely responsible for the cytoplasmic envelopment of tegument proteins and capsids in HCMV during the assembly and egress process [12,32].

## Tegument protein subcellular localization

As illustrated, the tegument proteins play important roles in all stages of the viral life cycle, including, viral entry, gene expression, immune evasion, assembly, and egress [11]. However, the subcellular localization of the tegument proteins after their release into the cytoplasm at the beginning stage of infection has not been fully elucidated. This is especially true for the localization of the tegument proteins when they are expressed individually or in combinations without the rest of the HCMV virion. Furthermore, the structure of the tegument itself is not known, and the process of assembling the tegument upon viral egress, as well as the disassembly of the tegument upon viral entry into cells, is poorly understood [17]. Therefore, the objective of this research was to determine the subcellular localization of the primary tegument proteins pp65, pp71, pp150, and pp28, after transfection of plasmid DNA expressing each protein as a fluorescent protein fusion. Since these tegument proteins play pivotal roles in several stages of the viral life cycle, knowledge of where and the mechanism of how these proteins localize upon release could be fundamental in the development of effective, novel antiviral treatments for this widespread human pathogen, which would have great therapeutic value for immunocompromised individuals.

When HCMV virions fuse with the membrane of host cells, some tegument proteins remain in the cytoplasm, while others migrate to the nucleus of the cell [33]. Other tegument proteins will remain tightly associated to the nucleocapsids, and mediate their delivery to the nuclear pore complex via the microtubule assembly as reviewed in Kalejta (2008) [12]. Several tegument proteins though will have a specific localization within the cell depending on the stage of the lytic cycle.

In the early stage of infection, pp65 tends to independently migrate to the nucleoli of the cell [34]. The localization of pp65 to the nucleoli suggests a functional relationship between the localization of pp65 and the development of the lytic cycle of HCMV [34]. However, pp65 begins to migrate to the cytoplasm 48 h into the lytic cycle with nuclear pore complex becoming devoid of the protein [35]. This migration appears to be mediated by cyclin-dependent kinase activity and a Crm1 exporter [36].

Pp71, by comparison, has a similar localization pattern as pp65 during the lytic cycle of HCMV infections. The subcellular localization of pp71 upon viral entry is to the nucleus of the host cell [33]. This localization appears to be essential for the initiation of either a lytic or latent infection [37]. During the later stages of infection, some pp71 appears to localize subcellularly to both the nucleus and the cytoplasm [38].

Unlike the subcellular localization of pp65 and pp71, which appears to be dependent on the stage of the lytic life cycle of HCMV, the localization of pp150 has not been well-defined. Some studies have suggested that the subcellular localization of pp150 initially when it associates with the viral nucleocapsids is to the nucleus, while other studies suggest that it is to the cytoplasm [39,40]. The subcellular localization of pp28 on the other hand appears to be to specific cytoplasmic compartments [41]. This localization appears to be essential for the production of viral progeny, since it is localized at the site of final envelopment [41].

## Fluorescence proteins as localization tags

The development of fluorescent protein molecules to act as localization tags for subcellular components has revolutionized the biomedical sciences [42]. The first instance occurred when *Aequorea victoria *jellyfish wild-type green fluorescent protein (GFP) was used to highlight sensory neurons in the nematode [43]. Fluorescence is such a powerful tool in that it allows one to distinguish and identify cellular components that are either too small or lack little contrast with the background with traditional microscopy techniques. The wild-type green fluorescent protein molecule was modified to yield different variants due to its complex emission spectrum [44]. Nonetheless, extensive research has occurred in recent years to produce new and improved fluorescent tags that are brighter, cover a broad spectral range, and also exhibit enhanced photostability, reduced oligomerization, pH insensitivity, and faster maturation rates [42].

Due to the success of various fluorescent protein molecules being used as tags to assess the subcellular localization and of many cellular and viral proteins, several distinct fluorescent protein molecules were utilized in this experiment to identify the subcellular localization of HCMV tegument proteins pp65, pp71, pp150, and pp28, respectively [44]. The fluorescent tegument protein fusions were constructed previously by inserting the open reading frame of each tegument protein into a plasmid containing a fluorescence gene so that the two protein coding regions are in-frame and produce a fusion protein (Pizzorno et al., unpublished data). After the plasmid and tegument proteins undergo restriction digests, the resultant sticky ends fuse to form the fluorescently labeled tegument protein plasmid. The fluorescent tegument protein fusions are identified by transforming them into bacterial cells on a plate containing a specific antibiotic. Since the plasmid contains the antibiotic resistance gene, the colonies that are transformed with the fusion plasmid are able to survive on the plate. The fusion plasmids are then isolated from the bacterial colonies that were transformed.

**Figure 2:**
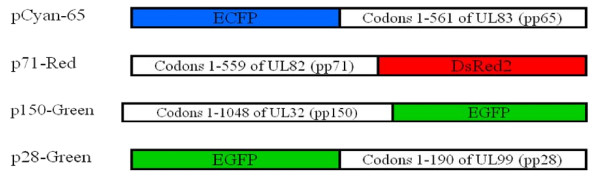
**Fluorescently labeled HCMV tegument protein constructs**.

In total, three variant fluorescent proteins (Cyan-blue, RFP (DsRed2)-red, GFP-green) were used to tag and label the tegument proteins. Pp65 was tagged with Cyan, pp71 with RFP, and pp150 and pp28 with GFP. The characterization of each fluorescent protein molecule can be seen in Table 1. Additionally, the location of the fluorescent tags on each protein can be seen in Figure 2.

**Table 1:** Characterization of the fluorescent molecules used to tag HCMV tegument proteins pp65, pp71, pp150, and pp28

Fluorescent Molecule	Tegument Protein(s) Labeled	Excitation Peak (nm)	Emission Peak (nm)	Brightness	Photostability	pKa
Cyan	pp65	433/445	475/503	13	64	4.7

RFP	pp71	584	607	12.5	8.7	4.5

GFP	pp150 and pp28	488	507	34	174	6.0

## Experimental outline and objectives [45]

As mentioned, the objectives of this study were to identify the subcellular localization of HCMV tegument proteins pp65, pp71, pp150, and pp28 to understand how these proteins interact with the cell and function during the lytic cycle of HCMV infections. Additionally, identification of the HCMV tegument protein localization patterns could assist in the development of a better targeted effective, novel antiviral treatment of HCMV infections, especially in immunocompromised individuals. This was accomplished through the use of cell culture and microscopy techniques. The tegument proteins of interest were first fused to a fluorescence protein (Cyan-blue, RFP-red, GFP-green), as described to in the previous section, that will emit/fluoresce when exposed to a certain wavelength of light. The DNA that encodes these modified tegument proteins were then transfected into an established human cell lines (HeLa cells). Confocal microscopy, which is an imaging technique that is used to increase the optical resolution and contrast of photographs obtained through a microscope, was then utilized to determine the subcellular localization of each tegument protein within the transfected cells by noting where the Cyan, RFP, or GFP molecule fluoresces to reconstruct three-dimensional structures of the obtained images. Additionally, the images obtained from the fluorescence tags were compared and overlaid with images obtained by concurrently staining with DAPI or Hoechst 33342, which are fluorescent stains that have a high affinity for DNA. Since the DNA is located within the nucleus of cells, this allowed for a definitive identification of where these tegument proteins localize subcellularly in transfected cells.

There were three distinct series of steps in determining the localization of the tegument proteins. The localization of the tegument proteins were first identified in fixed cells that were transfected with plasmids containing the tegument proteins of interest. Since the localization of each protein may be affected by the presence or absence of other tegument proteins, a series of transfections occurred, including the tegument proteins by themselves and a combination of the different tegument proteins of interest. Since we have three different fluorescence tags, we were able to observe the localization of three tegument proteins in a single cell. The localization patterns in the fixed cells were then compared to the same series of transfections in live cells to acquire more accurate observations of the localization patterns as well as to rule out the possibility that artifacts of the fixation process may have affected the subcellular localization patterns of the tegument proteins.

## Summary of results [45]

It was shown via fluorescent confocal microscopy that the localization of each tegument protein was independent of each other, and that live-cell imaging experiments gave better results than fixed cell imaging experiments. Furthermore, the subcellular localization of pp71 and pp150 (nucleus), and pp28 (cytoplasm) were identical to what occurs in a typical HCMV lytic infection, suggesting a strong correlation between localization and function. The most significant piece of information that can be drawn from this experiment, though, concerns that subcellular localization pattern that was observed for pp65. In the early stage of a normal HCMV infection, pp65 tends to independently migrate to the nucleoli of the cell [34]. This localization to the nucleoli suggests a functional relationship between the localization of pp65 and the development of the lytic cycle of HCMV [34]. In accordance with previous experiments, it was hypothesized that pp65 would subcellularly localize to the nucleus of the cell. However, it was seen that pp65 localizes to the cytoplasm of the transfected HeLa cells.

The observed localization pattern of pp65 suggests that something else in the HCMV virion, most likely another tegument protein, is crucial for pp65 to localize to the nucleus of a host cell in the early stages of infection. Moreover, the localization of pp65 to the nucleoli in the early stages of infection suggests a functional relationship between the localization of pp65 and the development of the lytic cycle of HCMV [24]. This implies that if a novel, antiviral treatment could target the other molecule in the HCMV virion that is required to assist pp65 to get into the nucleus of the host cell, HCMV infections and its devastating effects, especially in immunocompromised individuals, could be alleviated. This new data suggests that a potential effective, novel, antiviral treatment for HCMV infections could be synthesized from inhibiting the localization of pp65 to the nucleus of the cell in the early stages of infection.

The results that pp65 does not localize to the nucleus are interesting, since it is known that pp65 has a bipartite nuclear localization signal [46]. This implies that the mechanism by which the other molecule within the HCMV virion that is necessary for pp65 nuclear localization during the initial stages of the lytic infection is to expose the pp65 nuclear localization signal. It is known that proteins or other molecules with nuclear localization signals do not localize to the nucleus until the signal is exposed and recognized by an importin protein. Thus, the other molecule within the HVMC virion may bind to pp65, which yields a conformational change in pp65, thereby, exposing its nuclear localization signal to importin proteins during the early stages of the lytic cycle. Thus, a novel antiviral treatment could target the other molecule in the HCMV virion, and prevent pp65 from getting into the nucleus; subsequently inhibiting the HCMV lytic infection. This research has increased our understanding of HCMV lytic infections as well as identifies a potential target for a novel, antiviral treatment.

## Competing interests

The author declares that he has no competing interests.

## Authors' contributions

JT drafted and wrote the manuscript in addition to carrying out the cell based HCMV tegument protein transfections and subsequent fixed and live-cell confocal microscopy visualizations.

## Authors' information

John graduated as valedictorian from Mount Carmel Area Jr./Sr. High School in Mount Carmel, PA in 2007. He then attended Bucknell University in Lewisburg, PA where he earned a Bachelor of Science Degree with Honors in Cell Biology/Biochemistry in 2011. He is currently employed as a Biologist by Lancaster Laboratories in Lancaster, PA where he performs tissue culture based potency and viral quantification assays on live vaccine products. Additionally, he is employed by Fox Chase Cancer Center in Philadelphia, PA where he works as a Scientific Technician I in the lab of Dr. Matthew Robinson utilizing antibody-engineering techniques to develop molecularly targeted therapeutics designed to exploit the signal transduction networks that drive cancer formation and progression. He is looking to go on to get his PhD in either molecular biology, virology, immunology, or infectious diseases within the next few years.
